# Assessment of the impact of the scanner-related factors on brain morphometry analysis with Brainvisa

**DOI:** 10.1186/1471-2342-11-23

**Published:** 2011-12-21

**Authors:** Mahsa Shokouhi, Anna Barnes, John Suckling, Thomas WJ Moorhead, David Brennan, Dominic Job, Katherine Lymer, Paola Dazzan, Tiago Reis Marques, Clare MacKay, Shane McKie, Steven CR Williams, Stephen M Lawrie, Bill Deakin, Steve R Williams, Barrie Condon

**Affiliations:** 1 Department of Clinical Physics and Psychological Medicine, College of Medicine, Veterinary and Life Sciences, University of Glasgow, UK; 2 Brain Mapping Unit, Department of Psychiatry and Behavioural and Clinical Neurosciences Institute, University of Cambridge, UK; 3 Division of Psychiatry, Centre for Clinical Brain Sciences, School of Molecular and Clinical Medicine, University of Edinburgh, UK; 4 Department of Clinical Physics and Bioengineering, Institute of Neurological Science, Southern General Hospital, Glasgow, UK; 5 SFC Brain Imaging Research Centre, Division of Clinical Neurosciences, University of Edinburgh, UK; 6 Department of Psychosis Studies, Institute of Psychiatry, Kings College London, UK; 7 FMRIB Centre, University of Oxford, Oxford, UK; 8 Neuroscience and Psychiatry Unit, University of Manchester, UK; 9 Centre for Neuroimaging Sciences, Institute of Psychiatry, Kings College London, UK; 10 Imaging Science and Biomedical Engineering, University of Manchester, UK

## Abstract

**Background:**

Brain morphometry is extensively used in cross-sectional studies. However, the difference in the estimated values of the morphometric measures between patients and healthy subjects may be small and hence overshadowed by the scanner-related variability, especially with multicentre and longitudinal studies. It is important therefore to investigate the variability and reliability of morphometric measurements between different scanners and different sessions of the same scanner.

**Methods:**

We assessed the variability and reliability for the grey matter, white matter, cerebrospinal fluid and cerebral hemisphere volumes as well as the global sulcal index, sulcal surface and mean geodesic depth using Brainvisa. We used datasets obtained across multiple MR scanners at 1.5 T and 3 T from the same groups of 13 and 11 healthy volunteers, respectively. For each morphometric measure, we conducted ANOVA analysis and verified whether the estimated values were significantly different across different scanners or different sessions of the same scanner. The between-centre and between-visit reliabilities were estimated from their contribution to the total variance, using a random-effects ANOVA model. To estimate the main processes responsible for low reliability, the results of brain segmentation were compared to those obtained using FAST within FSL.

**Results:**

In a considerable number of cases, the main effects of both centre and visit factors were found to be significant. Moreover, both between-centre and between-visit reliabilities ranged from poor to excellent for most morphometric measures. A comparison between segmentation using Brainvisa and FAST revealed that FAST improved the reliabilities for most cases, suggesting that morphometry could benefit from improving the bias correction. However, the results were still significantly different across different scanners or different visits.

**Conclusions:**

Our results confirm that for morphometry analysis with the current version of Brainvisa using data from multicentre or longitudinal studies, the scanner-related variability must be taken into account and where possible should be corrected for. We also suggest providing some flexibility to Brainvisa for a step-by-step analysis of the robustness of this package in terms of reproducibility of the results by allowing the bias corrected images to be imported from other packages and bias correction step be skipped, for example.

## Background

Brain morphometry has proven to be a powerful tool in identifying biomarkers of many neurological and psychiatric disorders. Several studies have investigated the link between the changes in the brain morphology and certain diseases or disorders such as Alzheimer's disease, schizophrenia, Autism, Epilepsy, and Bipolar disorder [1-6].

One of the popular software packages for brain morphometry is Brainvisa [7]. In addition to the most common morphometry metrics, this program allows a sulcus-based morphometry. This is possible using the automatic sulci recognition feature of the program which automatically identifies the sulci of each individual brain. Sulcal parameters such as volume, depth, location, and pattern can then be computed for each sulcus. Exploring the sulcal parameters provides valuable information as it has been shown that changes in such parameters can be associated with pathology [5,8].

Brainvisa has been used for cortical morphometry and the abnormality-related changes in parameters such as sulcal mean depth and surface for patients with cerebral autosomal dominant arteriolopathy with subcortical infarcts and leukoencephalopathy (CADASIL) [9]. It has also been used to show the decrease in average cortical thickness and sulcal span with normal aging [10]. Moreover, decreased global sulcal index (GSI), the ratio between the folded surface and the unfolded surface of the cortex, has been reported for schizophrenic patients with auditory hallucinations as well as patients with bipolar disorder and unipolar depression [11-13].

Nonetheless, the reliability of the brain morphometry is a major issue in cross-sectional studies (evaluation of the differences between normal and abnormal brains), where the abnormality-related variation may be small and dominated by the low measurement reliabilities.

Scanner-related factors can complicate the cross-sectional studies where both between-group variability and within-group variability (due to inter-individual variability in brain anatomy) already exist. Scanner instability and variations over time may result in bias in the derived morphometric measures as already shown for functional MRI and should be taken into account especially in longitudinal studies such as normal brain aging [14]. Furthermore, there has been a growing interest in multicentre studies as they provide the researchers with larger datasets by pooling data from different sites and hence improve the statistical power [15,16]. Nevertheless, multicentre studies may introduce a between-centre variance component which can overshadow small between-group (patients vs. normal subjects) variances. Consequently it is essential to verify the effect of scanner-related factors (either within-centre or between-centre) on the estimated values for the morphological parameters.

While a considerable amount of studies on the scanner-related variability have focused on functional MRI [17-19], a number of similar studies concerning structural MRI have also been reported. Using their previously developed algorithm, Schnack et al. studied the variability of brain tissue segmentation for data acquired from multiple centres, different manufacturers and under different acquisition protocols [20,21]. Han et al. looked into the effects of scanner-related factors such as field strength, scanner manufacturer, upgrade, and pulse sequence as well as data processing factors on the cortical thickness measurement using FreeSurfer [22,23]. Moorhead et al. investigated both within-scanner and between-scanner variability in the segmentation of grey and white matters using SPM5 [24,25]. Suckling et al. studied both within-centre and between-centre variability in the distribution of grey matter using FSL with the aim of power calculation for two-group, cross-sectional study designs [26,27]. The above studies have considered some of the currently used morphometric measures, however there has been no similar reports for the automatically computed sulcal attributes despite being used in morphometry analysis. More research regarding such measures still needs to be carried out.

In addition, the measurement process introduces another source of variance which may vary among different packages. Thus, the reliability also depends on the measurement method and the package used for the analysis. Nonetheless, a study on the assessment of morphometry with Brainvisa has not been reported yet. This paper presents a comprehensive study on the assessment of reliability and robustness of morphometry using Brainvisa against the scanner-related variability, whilst also investigating the possible causes which reduce the reliability. Our aim in this study has been twofold: 1) assessment of viability of multicentre and longitudinal studies using Brainvisa and 2) investigating the robustness and reproducibility of morphometry with Brainvisa using repeated scans (both between- and within-centre) of the same subjects.

To cover the most commonly used morphological parameters, we estimated brain tissue volumes and GSI as well as the sulcal attributes. For sulcal attributes, the assessments were performed independently with each of the four recognition algorithms provided in Brainvisa, as they produce slightly different results. The above-mentioned choice of parameters is also useful in the assessment of the reliability associated with each particular pre-processing step within Brainvisa. To further investigate the possible causes which could have an impact on the reliability, brain segmentation was repeated using FSL and the results were compared to those obtained with Brainvisa.

Moreover, to verify the performance of Brainvisa in different field strengths (and hence various degrees of signal to noise ratio) we used two separate groups of data acquired with 1.5 T and 3 T scanners and investigated the variability and the reliability within each group.

## Methods

### Data

The retrospective data we used in this study included two sets of 3D T1-weighted MR scans, pooled from 1.5 T and 3 T scanners of CaliBrain (funded by a Chief Scientist Office -Scotland Project Grant: CZB/4/427) and Neuro/Psygrid projects, respectively [28,29]. Both datasets had been obtained from healthy individuals with no history of head injury, psychiatric or neurological disorder. Two subjects (one from the CaliBrain dataset and one from the Neuro/Psygrid dataset) with incomplete data (i.e. repeated scans at all centres) were excluded from the study. The study was approved and the permission to use the retrospective data was granted by the West of Scotland Research Ethics Committee.

The CaliBrain project included MR scans from thirteen healthy participants (ten male, mean age 36.3, age range 22-51 years). The subjects had been scanned twice at three different sites: The Department of Radiology, University of Aberdeen; The Division of Psychiatry and The SFC Brain Imaging Research Centre within The Centre for Clinical Brain Sciences (CCBS) at The University of Edinburgh; and The Institute of Neurological Sciences, NHS Greater Glasgow South University Hospitals Division. The T1-weighted scans were acquired using a 3D inversion recovery-prepared fast gradient echo volume sequence. All three 1.5 T scanners were manufactured by General Electric (GE Healthcare, Milwaukee, Wisconsin).

For the Neuro/Psygrid project, eleven male, healthy volunteers (mean age 25, range 20-35 years) were scanned twice at five centres: The Wolfson Brain Imaging Centre (University of Cambridge), Magnetic Resonance Imaging Facility (University of Manchester), the Institute of Psychiatry (Kings College, London), the Department of Clinical Neurosciences at the University of Edinburgh and the Institute of Neurological Sciences in Glasgow, and the Centre for Clinical Magnetic Resonance Research, (University of Oxford). Of all the MR scanners, two were manufactured by GE, two by Siemens, and one by Philips. The 3D T1-weighted images were acquired using the Magnetization Prepared Rapid Gradient Echo (MP-RAGE) sequence [30]. Briefly, our whole dataset consisted of two groups: the first group included thirteen subjects scanned twice at three centres with 1.5 T scanners (presented here as A, B, and C) and the second group consisted of eleven subjects scanned twice at five centres using 3 T scanners (presented here as D, E, F, G, and H). The scanner type and manufacturer as well as imaging parameters for the two groups of datasets are given in tables 1 and 2. In all cases, the first and second visits were performed using the same scanner. Only one volume was acquired at each scanning session.

**Table 1:** The scanners specifications and imaging parameters for the 1.5 T group

Centre	A	B	C
MR scanner	GE Signa NVi/CVi 1.5 T	GE Signa LX 1.5 T	GE Signa 1.5 T

Head coil	8-channel	8-channel	8-channel

Acceleration factor	No	No	No

TR (ms)	5.9	8.9	5.9

TE (ms)	1.9	3.3	1.4

TI (ms)	600	600	600

Flip angle (°)	15	15	15

Image size (voxels)	256 × 256 × 124	256 × 256 × 124	256 × 256 × 125

Voxel size (mm^3^)	0.86 × 0.86 × 1.7	0.86 × 0.86 × 1.7	0.86 × 0.86 × 1.8

TR, TE, and TI represent Repetition Time, Echo Time, and Inversion Time, respectively.

**Table 2:** The scanners specifications and imaging parameters for the 3 T group

Centre	D	E	F	G	H
MR scanner	GE 3 T HD	GE 3 T HDx	Siemens 3 T Tim Trio	Siemens 3 T Tim Trio	Philips 3 T Intera-Achieva

Head coil	8-channel	8-channel	8-channel	12-channel	8-channel

Acceleration factor	No	ASSET factor 2	No	No	SENSE factor 2

TR (ms)	6.5	7.0	9.0	9.4	8.2

TE (ms)	1.50	2.85	2.98	4.66	3.80

TI (ms)	500	650	900	900	885

Flip angle (°)	12	8	9	8	8

Image size (voxels)	260 × 260 × 160	260 × 260 × 160	256 × 256 × 160	256 × 256 × 160	256 × 256 × 160

Voxel size (mm^3^)	1.1 × 1.1 × 1	1.1 × 1 × 1	1 × 1 × 1	1 × 1 × 1	1 × 1 × 1

TR, TE, and TI represent Repetition Time, Echo Time, and Inversion Time, respectively.

### Data pre-processing

**Figure 1:**
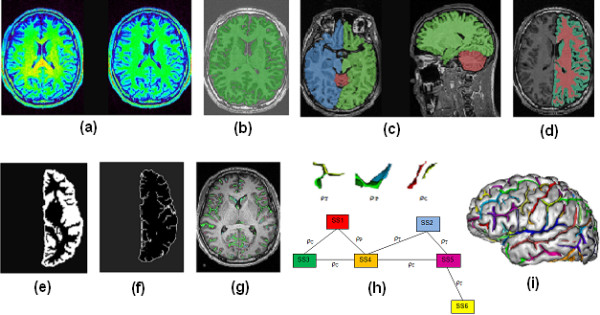
**Different steps of data pre-processing in Brainvisa**. a) a colour-coded presentation of the raw (left) and bias corrected (right) T1-weighted images b) the extracted brain overlaid on the T1 image c) the brain split into the right and left hemispheres as well as the cerebellum d) each hemisphere segmented into GM, WM and CSF e) the union of GM and CSF f) the GM/CSF union following skeletonization g) the skeleton surface on the T1-weighted image after removing the brain hull h) the three possible relations between neighbouring folds (top) and a graph symbolizing the folds and their mutual relations (bottom) i) colour-coded presentation of the identified sulci. The figure does not correspond to the data used in this study and is presented only for illustration purposes.

Pre-processing of the 3D T1 images was carried out using the segmentation pipeline of Brainvisa (version 4.0) and included the following steps (also illustrated by Figure 1): The 3D T1 image was corrected for the field inhomogeneity (bias correction) so as to obtain more uniform grey levels for all voxels with the same tissue type [31]. Figure 1a shows a colour-coded representation of the T1 image before (left) and following (right) the bias correction. After this step, the image is ready for histogram analysis which finds the peaks corresponding to grey matter (GM), white matter (WM), and cerebrospinal fluid (CSF) in the image histogram [32]. Then a mask of brain was created using the information derived from histogram analysis and by applying morphological operations which removed skull and non-brain tissues (Figure 1b). The two hemispheres and the cerebellum were then separated (Figure 1c) and further segmented into GM, WM, and CSF as pictured by Figure 1d[33]. The union of GM and CSF (GM/CSF union) was found (Figure 1e) and then skeletonised (Figure 1f). The skeleton points connected to the outside representing the brain hull were then removed. Figure 1g shows the remaining points of the skeleton on the T1 image. Afterwards the skeleton was divided into simple surfaces, i.e. pieces of surfaces that do not contain any junctions. Each simple surface represents a cortical fold. Each simple surface was then split further to represent situations where a gyrus has been buried in the bottom of the fold. The folds and their mutual relationships were finally gathered in a graph [34]. Figure 1h shows a schematic explanation of the possible relationships between the related folds and how this information is summarized in the graph. Three kinds of relations exist: topological junction (ρ_T_), split induced by a buried gyrus, called a "pli de passage", (ρ_P_), and neighbour geodesic to the brain hull (ρ_C_). Each node of the graph (fold) is specified by certain attributes including the links with other nodes. Figure 1h also exemplifies how the graph nodes (shown as SS for Simple Surface) can be linked through any of the three possible relationships. Several folds were then grouped to form each sulcus using prior information from the probabilistic location of that sulcus. The process of sulci recognition is discussed in more detail in the next section. Figure 1i displays a colour-coded presentation of the recognized sulci on the reconstructed mesh of the brain where each colour corresponds to a sulcus.

The outputs of bias correction, brain extraction and split, and segmentation steps were visually examined and were corrected by tuning the parameters and repeating the process where necessary. In one or two cases, manual corrections were used to produce reasonable results.

### Sulci recognition

The folds gathered in the relational graph structure were grouped together to form the sulci. The sulci recognition algorithm uses prior knowledge about the location of each sulcus for labelling the folds. Sulci labelling with Brainvisa is based on the sulcal root theory and returns 59 sulcal labels for each hemisphere [35].

For sulci recognition we used the Statistical Parametric Anatomy Map (SPAM) algorithm which uses a probabilistic model as the a priori information for sulci recognition. This probabilistic model returns the probability of presence of each sulcus at a given 3D position [36,37]. There are four variations of the SPAM algorithms: Talairach, Global, Local, and Markovian. The Talairach method uses the probabilities of sulci locations in the Talairach space. However, as the sulci alignment of different subjects is not accurately achieved by registering the brain to the Talairach atlas, the other three algorithms use three approaches to improve between-individual sulcal alignment. The Global approach is based on iterative registration and labelling of the cortical folds to the SPAM maps where the same registration parameters are applied to the whole cortical surface. The Local method which is performed following a Global registration locally optimizes the registration on a sulcus-by-sulcus basis; hence each sulcus has its unique registration parameters. The Markovian algorithm also follows the Global registration and uses the information about the relations and distances between neighbouring sulcal segments for labelling the sulci. It should be noted that sulci recognition involves a virtual registration to a template and the final results (including sulcal parameters) are expressed in the native space.

For this study we employed all four algorithms independently. This allowed us to estimate the sulcus-specific morphometric measures for each method and compare the reliability obtained by each method.

### Morphometry analysis

Using Brainvisa, the morphometric measures were calculated for every scan of each subject. The measurements were performed for each cerebral hemisphere independently (Figure 1d). The morphometric measures were either global (brain tissue volumes, and global sulcal index) or sulcal (parameters that were calculated for each sulcus independently; i.e. sulcus surface and sulcus mean geodesic depth).

### Global parameters

Brain tissues volumes: Brain segmentation (Figure 1d) was used to estimate the volumes of WM, GM, and CSF for each hemisphere.

Global Sulcal Index (GSI): This is an estimation of the cortex gyrification and is defined as the ratio between the total area of all the cortical folds and the area of the outer cortical surface (unfolded cortex).

### Sulcal parameters

The following sulcal parameters were calculated for each of the four SPAM algorithms separately:

Sulcus surface: Since the sulci are formed from the skeleton segments (Figure 1g) which are only one voxel thick, their volumes depend on the voxel size and orientation. Instead, the surface area of the sulcus which is not affected by voxel size provides a better estimate of sulcus size. Therefore we used sulcus surface area as the proxy for its volume.

Sulcus mean geodesic depth: The geodesic depth of sulcus is defined as the geodesic distance (along the cortical mesh) between the external line of the fold (on the brain hull), and the bottom line of the sulcus.

### Statistical analysis

For each morphometric measure, Analysis of Variance (ANOVA) was conducted using Minitab 16 at the significance level of 0.05 to investigate whether the values corresponding to different centres or different visits were significantly different. Thus an effect was considered significant if the observed p-value under the null hypothesis that the effect is non-significant was smaller than 0.05. P-values were not adjusted for multiple testing and therefore have to be considered as descriptive. In order to take the between-subject difference into account, a "subject" factor was included in the ANOVA model. For the analysis of GM, WM, and CSF volumes as well as GSI, the brain volume was also included as covariate. For sulcal parameters, both brain volume and GSI were included as covariates.

In addition to the significance of centre and visit factors, we evaluated the scanner-related reliability to assess the influence of the variability on the ability to distinguish between individuals. In other words, the reliability gives an estimate of how close the values calculated from different scans of the same subject are and whether the between-centre or between-visit variability is the dominant source of variation which overshadows the between-individual variability. For this purpose we used a random effects ANOVA model and computed the variance components from all sources of variability across different centres and visits of each field strength group. The reliability associated with each factor was then calculated as the ratio of the variance excluding the contribution from that factor to the total variance.

(1) $$\begin{array}{c} \text{between}-\text{centre reliability}=\frac{{\text{V}}_{\text{total}}-{\text{V}}_{\text{centre}}}{{\text{V}}_{\text{total}}}\\ \text{between}-\text{visit reliability}=\frac{{\text{V}}_{\text{total}}-{\text{V}}_{\text{visit}}}{{\text{V}}_{\text{total}}} \end{array}$$

Where V_total _is the total variance corresponding to all sources of variability and is obtained from the sum of all variance components, and V_centre _(or V_visit_) is the sum of the variances associated with the centre (or visit) factor and its interactions with other factors. The reliability ranges from zero to one, representing cases where the variance associated with the centre (or visit) factor is the only source of variance or is negligible, respectively.

### Segmentation with FSL vs. Brainvisa

In order to further explore the factors limiting the reliability of Brainvisa morphometry we used FSL (version 4.1.4) and compared the results of the two packages. Since bias correction and histogram analysis are the fundamental steps with great influence on the final results, a comparison between the two packages in brain segmentation (which is a direct result of these two steps) can be helpful in the assessment of robustness of each of these processes.

While it is usually suggested to use SIENA/SIENAX tools within FSL for longitudinal or cross-sectional studies, these tools may introduce bias since they involve a registration step. Consequently, we chose the FAST algorithm (version 4.1) of FSL which employs a Hidden Markovian Random Field model that for voxel classification, takes the tissue type of its neighbouring voxels into account, whilst also correcting for the bias field [38]. To ensure that the comparison between the two packages only concerns the segmentation process and not the brain extraction, the same skull-stripped brain created with Brainvisa was used and segmented using FAST.

Two metrics were used to compare the bias correction of FAST and Brainvisa: the coefficient of variation within each tissue type, and the WM/GM contrast. More robust bias correction should result in smaller values for the coefficient of variation (due to narrower histogram peaks) and higher WM/GM contrast. Masks of all three tissue types were applied to each bias corrected T1 image (bias corrected with Brainvisa or FAST) and the coefficient of variation was calculated within each mask. To avoid bias towards either of the two programs, each tissue mask was defined as the intersection of the masks obtained with FAST and Brainvisa. To estimate the WM/GM contrast, the WM and GM peaks were computed from the distribution of grey levels within the WM and GM masks. The WM/GM contrast was then calculated as the ratio between the WM and GM peaks. It should be noted that this method of peak detection is different and independent from peak detection with the histogram analysis in Brainvisa.

## Results

**Figure 2:**
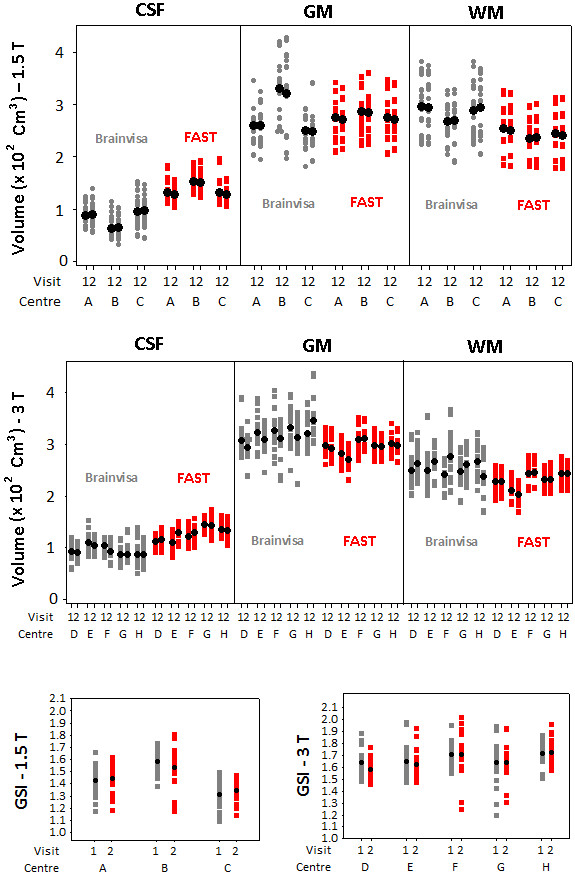
**Plots of the distribution of global parameters across subjects**. The volumes of CSF, GM, and WM are calculated using FAST and Brainvisa for the two visits to all centres of the 1.5 T group (top) and the 3 T group (middle). The plots of GSI for the 1.5 T groups (bottom left) and the 3 T group (bottom right) are also shown. The values corresponding to both hemispheres are presented (each value corresponds to one hemisphere). The mean values have also been shown in each individual plot. Labels 1 and 2 refer to the first and second visits, respectively. The statistical results of this figure can be found in tables 5 and 8.

The distribution of the global parameters across all subjects, visits, and centres are displayed in Figure 2. The volumes of GM, WM, and CSF calculated by Brainvisa and FAST have been shown. This figure also demonstrates the GSI values. The values have been calculated for each hemisphere independently and thus each value corresponds to only one hemisphere. The numerical presentation of segmentation results with Brainvisa and FAST are also given in tables 3 and 4 for the 1.5 T and 3 T groups, respectively.

**Table 3:** The mean and standard deviation of GM, WM, and CSF volumes across subjects for the 1.5 T group

Centre/Visit	GM volume (cm^3^)		WM volume (cm^3^)		CSF volume (cm^3^)	
	Brainvisa	FAST	Brainvisa	FAST	Brainvisa	FAST
A/1	259 ± 35	275 ± 36	296 ± 45	253 ± 44	87 ± 18	132 ± 20

A/2	259 ± 34	270 ± 32	293 ± 47	249 ± 39	89 ± 20	127 ± 16

B/1	322 ± 65	286 ± 34	274 ± 29	234 ± 33	62 ± 21	153 ± 18

B/2	321 ± 68	285 ± 35	274 ± 24	236 ± 34	64 ± 19	151 ± 21

C/1	249 ± 31	274 ± 38	288 ± 48	243 ± 41	96 ± 28	131 ± 23

C/2	247 ± 34	270 ± 32	293 ± 51	239 ± 38	96 ± 26	128 ± 15

The results of segmentation using Brainvisa and FAST are given (mean ± standard deviation) for each visit to each centre

**Table 4:** The mean and standard deviation of GM, WM, and CSF volumes across subjects for the 3 T group

Centre/Visit	GM volume (cm^3^)		WM volume (cm^3^)		CSF volume (cm^3^)	
	Brainvisa	FAST	Brainvisa	FAST	Brainvisa	FAST
D/1	308 ± 31	298 ± 20	249 ± 29	228 ± 20	93 ± 17	112 ± 12

D/2	294 ± 25	293 ± 19	264 ± 28	227 ± 18	91 ± 14	116 ± 15

E/1	324 ± 28	282 ± 17	250 ± 42	211 ± 19	111 ± 21	111 ± 15

E/2	310 ± 27	271 ± 20	268 ± 31	202 ± 17	105 ± 11	130 ± 16

F/1	328 ± 36	309 ± 22	241 ± 23	245 ± 21	105 ± 11	122 ± 15

F/2	311 ± 32	312 ± 21	278 ± 43	246 ± 22	94 ± 13	129 ± 14

G/1	332 ± 39	298 ± 18	247 ± 28	232 ± 16	87 ± 18	144 ± 13

G/2	314 ± 37	295 ± 19	261 ± 28	232 ± 18	88 ± 18	143 ± 18

H/1	321 ± 30	301 ± 17	267 ± 34	245 ± 20	87 ± 26	136 ± 13

H/2	346 ± 38	299 ± 18	237 ± 34	244 ± 19	87 ± 17	133 ± 14

The results of segmentation using Brainvisa and FAST are given (mean ± standard deviation) for each visit to each centre

As Figure 2 and table 3 suggest, for the 1.5 T group the distribution and the mean values are relatively consistent between the two visits with both Brainvisa and FAST. However, there is less consistency between centres and this inconsistency is more pronounced with Brainvisa. More specifically, the values estimated for centre B seem to considerably vary from those of the other two centres.

For the 3 T group with both methods, the between-visit consistency is less compared to the 1.5 T group (compare to the statistical results given in table 5).

**Table 5:** The p-values for centre and visit effects

Parameter	1.5 T Group			3 T Group		
	Centre	Visit	Centre*visit	Centre	Visit	Centre*visit
GM volume	< 0.001	0.272	0.875	< 0.001	0.015	< 0.001

WM volume	< 0.001	0.624	0.642	0.036	< 0.001	< 0.001

CSF volume	0.013	0.311	0.993	< 0.001	0.003	0.535

Cerebral	< 0.001	0.739	0.285	< 0.001	0.599	0.109
Hemisphere						
Volume						

GSI	0.017	0.011	0.024	0.203	0.088	0.094

Sulcal surface						
Global	0.827	0.895	0.885	< 0.001	0.777	0.843
Local	0.821	0.909	0.808	< 0.001	0.673	0.807
Markovian	0.778	0.855	0.944	< 0.001	0.486	0.865
Talairach	0.847	0.792	0.925	< 0.001	0.847	0.863

Sulcal mean						
geodesic depth						
Global	0.887	0.754	0.547	0.028	0.937	0.649
Local	0.573	0.820	0.574	0.002	0.121	0.943
Markovian	0.742	0.422	0.603	0.001	0.182	0.381
Talairach	0.574	0.427	0.857	0.006	0.956	0.711

P-values for the main effect of centre and visit as well as their interaction for all Brainvisa metrics are given for the 1.5 T and 3 T groups. For sulcal parameters, p-values are given for all sulci recognition algorithms.

For both groups, on average, Brainvisa seems to be classifying more voxels as WM compared to FAST. Figure 2 also shows that within the 1.5 T group, the GSI values corresponding to the two visits to centre B are less consistent compared to those for centres A or C. This observation can also be confirmed from table 5 which shows that the interaction of centre and visit is significant (p = 0.024), indicating that the between-visit variability for centre B is significantly different for those for centres A and C. The mean GSI was equal to 1.44 and 1.66 for the 1.5 T and 3 T groups, respectively. As GSI presents the ratio of the buried to unburied cortex, the fraction of the whole cortex that is buried in sulci is equal to GSI/(1+GSI). Thus on average, it has been estimated that for the 1.5 T group 59% and for the 3 T group 62% of the cortex is buried in sulci.

**Figure 3:**
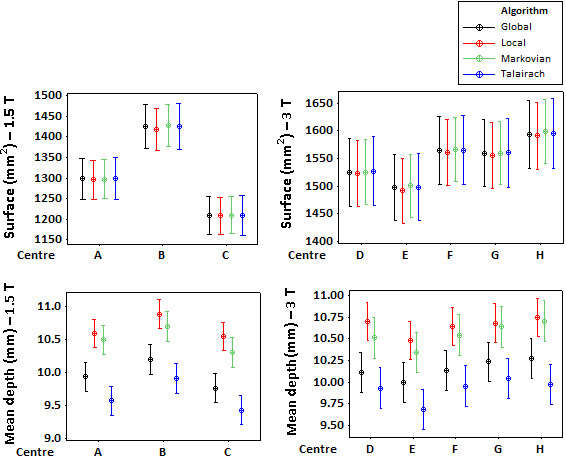
**The mean and 95% confidence interval of the sulcal parameters**. The mean (the average of all subjects, visits, and sulci) and the 95% confidence interval of the sulcal surface (top) and mean geodesic depth (bottom), calculated for each centre using all sulci recognition algorithms are shown for the 1.5 T group (left) and the 3 T group (right). Mean geodesic depth refers to the average geodesic depth across a given sulcal label (note that the depth a sulcus is usually non-uniform throughout its length and hence the mean value is used). The values are calculated for each hemisphere independently and the plots show the values associated with both hemispheres. The statistical results corresponding to this figure can be found in table 5.

Figures 3 gives an overview of the mean values and the 95% intervals for the sulcal surface and mean geodesic depth with all four sulci recognition algorithms. This figure shows qualitatively that all algorithms are very consistent in predicting the mean and the 95% confidence interval for the sulcus surface at each centre. However, the mean geodesic depth is estimated differently and on average, Local and Markovian algorithms predict higher values for the mean geodesic depths compared to Global and Talairach algorithms.

### Visit and centre effects

The p-values of the main effects for centre and visit as well as their interaction are given in table 5 for all morphometric measures. For sulcal parameters, the p-values were computed for all recognition algorithms.

The volume of cerebral hemisphere was calculated from the sum of GM, WM, and CSF for each hemisphere of each subject's brain. The comparison between the results for the hemisphere volume and those for the GM, WM, and CSF volumes allows for the assessment of robustness of the segmentation process.

As shown in table 5, with the exception of GSI for the 1.5 T group and GM and WM volumes for the 3 T group, for all other parameters the interaction between the visit and centre factors was non-significant. The significance of the interaction term is indicative of inconsistency in the between-visit variability across different scanners. There are two possible situations which can lead to such inconsistency and therefore significance of the interaction between centre and visit factors: 1) compared to the first visit to each centre, the values estimated with the second visit are higher for some scanners but lower for others, or 2) for some scanners, the two visits produce similar results whereas for other scanners the estimations for the two visits are significantly different. As visits don't follow any logical order, the significance of the interaction term in the first case may not necessarily be indicative of real inconsistency between scanners. This seems to be the case for GM and WM volumes of the 3 T group. In this case, as can be inferred from Figure 2, the direction of change from the first to the second visit varies across scanners. Conversely, for GSI of the 1.5 T group the second explanation applies as the significance of the interaction terms arises from higher between-visit variability for centre B compared to those for centres A and C.

Table 5 also indicates that for the 1.5 T group, the two visits to the same centre did not produce significantly different values for GM, WM, CSF, and hemisphere volumes, however these values were significantly different across centres.

In the 3 T group, although the two visits produced similar results for hemisphere volume, the GM, WM, and CSF volumes varied significantly between the two visits. In addition, all volumes significantly vary across scanners. Nonetheless, both visits to all five centres produced similar results for GSI.

For sulcal parameters, the centre and visit factors as well as their interaction were non-significant for the 1.5 T group, however for the 3 T group, the centre factor was significant for both surface and depth and with all four algorithms.

### Between-visit and between-centre reliabilities

The between-visit and between-centre reliabilities were computed for all parameters according to equation 1. As a rule of thumb, the reliability values smaller than 0.50, between 0.50 and 0.70, between 0.70 and 0.90, and greater than 0.90 were considered poor, moderate, good, and excellent, respectively.

Table 6 shows the results for global parameters. In the 1.5 T group, the between-visit reliability was excellent for the GM, WM, CSF and hemisphere volumes. Between-centre reliabilities were excellent for hemisphere volume, moderate for WM and CSF volumes, and poor for GM volume. For GSI, the between-visit reliability was good whereas between-centre reliability was poor.

**Table 6:** The between-visit and between-centre reliabilities for global parameters calculated using Brainvisa

Parameter	1.5 T Group		3 T Group	
	Between-visit	Between-centre	Between-visit	Between-centre
GM volume	0.92	0.36	0.29	0.25

WM volume	0.95	0.67	0.13	0.15

CSF volume	0.90	0.50	0.55	0.34

Cerebral hemisphere volume	0.99	0.97	0.96	0.88

GSI	0.75	0.13	0.62	0.43

The results are given for both 1.5 T and 3 T groups.

For the 3 T group, the between-centre and between-visit reliabilities of the hemisphere volume were almost excellent. However following segmentation, the reliabilities of the resulting segmented volumes were mostly poor. It can be therefore concluded that segmentation has led to a significant reduction in the reliability. The between-visit and between-centre reliabilities of GSI for the 3 T group were moderate and poor, respectively.

**Figure 4:**
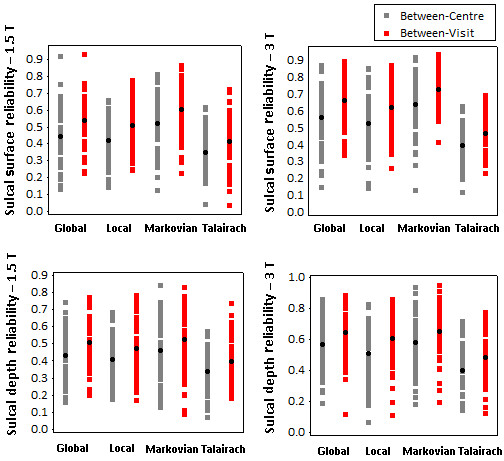
**Distribution of between-centre and between-visit reliabilities for sulcal parameters**. The distribution of the reliability of sulcal surface (top) and mean geodesic depth (bottom) is displayed for different sulci using all sulci recognition algorithms. The results correspond to the 1.5 T group (left) and the 3 T group (right).

The reliabilities for sulcal parameters were calculated for each sulcal piece independently due to huge variability in sulcal parameters (especially the sulcal surface) for different sulci. Figure 4 depicts the distribution of reliabilities across 59 sulcal pieces using all sulci recognition algorithms indicating that the reliabilities range from poor to excellent. The figure suggests that in all cases the average between-visit reliability was greater than the average between-centre reliability. Moreover among the four algorithms, Markovian and Global resulted in the highest reliabilities, whereas Talairach led to the lowest reliabilities. For a quantitative comparison between the four algorithms, the average reliabilities (across all sulci) are presented in table 7 for both 1.5 T and 3 T groups.

**Table 7:** The average between-visit and between-centre reliabilities of sulcal parameters

Parameter	1.5 T		3 T	
	Between- visit	Between- centre	Between- visit	Between- centre
Surface				
Global	0.54	0.44	0.66	0.56
Local	0.51	0.42	0.62	0.52
Markovian	0.60	0.52	0.73	0.64
Talairach	0.41	0.35	0.47	0.39

Mean geodesic depth				
Global	0.51	0.43	0.65	0.57
Local	0.47	0.41	0.61	0.51
Markovian	0.52	0.46	0.66	0.58
Talairach	0.40	0.34	0.48	0.40

The results are given as the average across all sulci and are computed for the 1.5 T and 3 T groups using all four algorithms.

### Segmentation with FAST vs. Brainvisa

The p-values of the centre and visit factors as well as their interaction for GM, WM, and CSF volumes estimated using FAST are given in table 8. For the 1.5 T group, each segmented volume was significantly different across centres but similar for the two visits to the same scanner. The interaction term was significant for WM volume which was mainly due the various directions of changes from first to second visit (as can be concluded from Figure 2).

**Table 8:** The p-values for segmentation with FAST

Parameter	1.5 T Group			3 T Group		
	Centre	Visit	Centre*visit	Centre	Visit	Centre*visit
GM volume	0.054	0.868	0.651	< 0.001	< 0.001	< 0.001

WM volume	< 0.001	0.230	0.028	< 0.001	< 0.001	< 0.001

CSF volume	0.018	0.312	0.311	< 0.001	< 0.001	< 0.001

P-values for the main effect of centre and visit as well as their interaction are given for the 1.5 T and 3 T groups.

For the 3 T group, the centre and visit factors as well as their interaction were significant for all volumes which suggests that some scanners produce less consistent results between the two visits. Figure 2 shows that this inconsistency mainly arises from centre E.

Table 9 summarizes the between-visit and between-centre reliabilities of brain segmentation using FAST. The table suggests that except for CSF, the reliabilities of GM and WM volumes have significantly improved with FAST.

**Table 9:** The between-visit and between-centre reliabilities for segmentation with FAST

Parameter	1.5 T Group		3 T Group	
	Between-visit	Between-centre	Between-visit	Between-centre
GM volume	0.88	0.84	0.72	0.47

WM volume	0.93	0.89	0.77	0.41

CSF volume	0.78	0.49	0.65	0.22

The results are given for both 1.5 T and 3 T groups.

**Figure 5:**
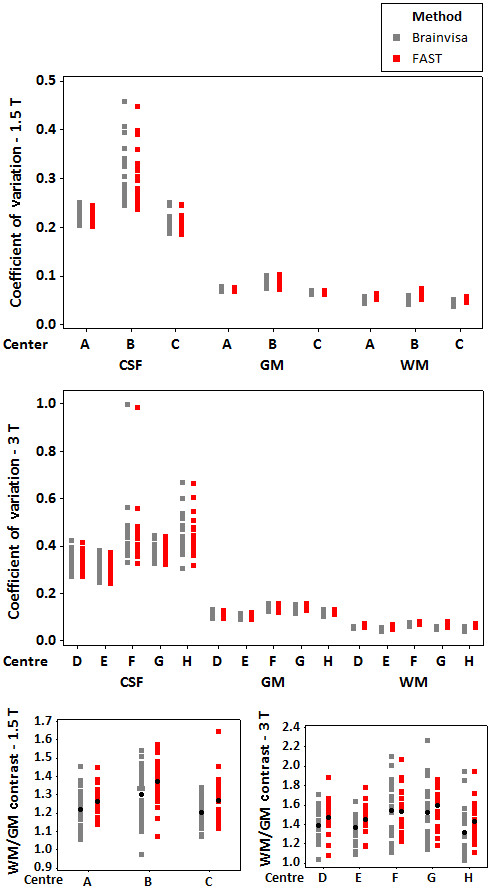
**Comparison of bias correction using Brainvisa and FAST**. The coefficient of variation within the CSF GM, and WM masks of the bias corrected T1 images are presented for the 1.5 T (top) and the 3 T (middle) groups. The WM/GM contrasts are shown for the 1.5 T (bottom left) and 3 T (bottom right) groups. Since the FAST algorithm does not split the cerebrum into two hemispheres, the values are calculated within both hemispheres (and not individual hemispheres) for both Brainvisa and FAST algorithms.

**Figure 6:**
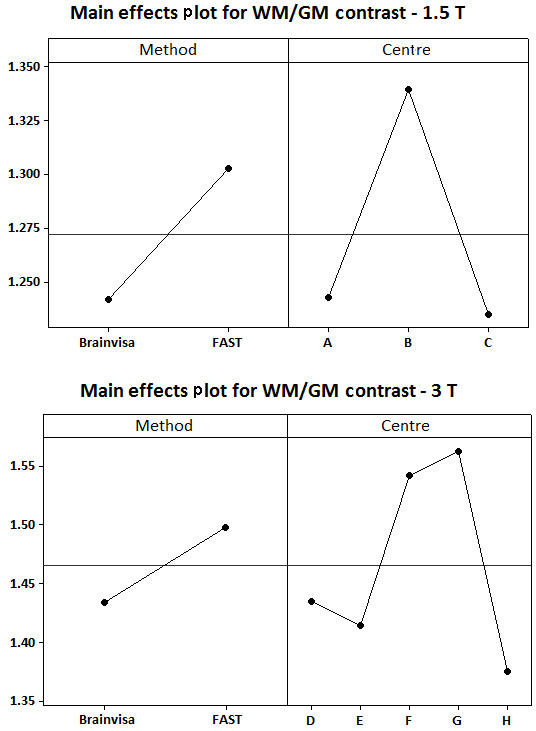
**Plots of main effect of Centre and Method for the WM/GM contrast**. The main effect plots are shown for the 1.5 T (top) and 3 T (bottom) groups.

Figure 5 demonstrates the effect of bias correction with Brainvisa and FAST in terms of the coefficient of variation within each tissue type as well as the WM/GM contrast for the 1.5 T and 3 T groups. As can be seen, the calculated coefficients of variation with FAST and Brainvisa were very similar. According to the figure, the coefficient of variation was lowest within WM and slightly higher within GM which is mainly due to the partial volume effect and considerably higher for CSF [31]. Figure 5 suggests that with FAST there's significant increase in WM/GM contrast compared to Brainvisa (p-value of smaller than 0.001 for 1.5 T group and 0.032 for 3 T group). This is also confirmed by Figure 6 which shows the plots of the main effects for method (Brainvisa vs. FAST) and centre for the WM/GM contrast. The figure shows that for the 1.5 T group, centre B had higher WM/GM contrasts compared to centres A and C. For the 3 T group, centres F and G had highest values and centre H had the lowest values of WM/GM contrasts.

## Discussion

We have assessed the robustness of Brainvisa in brain morphometry and estimation of the most widely used morphometric measures in terms of scanner-related variability and reliability. For this purpose we used two groups of retrospective datasets from two multicentre studies which included repeated scans acquired at 1.5 T and 3 T from healthy volunteers.

In some cases the morphometry results were significantly different across different scanners or across the two visits to the same scanner. It should be noted however, that the results of this study correspond to small groups of subjects and therefore for a larger dataset, even the non-significant cases may become significant. In addition, both between-centre and within-centre reliabilities ranged from poor to excellent for most parameters which also emphasizes the impact of scanner-related factors. However the within-centre reliability was found to be better than the between-centre reliability for almost all morphometric measures.

A comparison between the reliability values for the cerebral hemisphere volume and the segmented tissues (GM, WM, and CSF) revealed that while hemisphere volumes were very consistent both between- and within-scanners, the segmented volumes showed considerably different result in most cases. This implies that the inconsistency between the brain tissue volumes had arisen from the segmentation process. When the segmentation was carried out using the FAST algorithm within FSL, the reliabilities were mostly improved. Further investigation indicated that despite comparable values for the coefficients of variation within each tissue obtained with the two methods, FAST resulted in significantly higher WM/GM contrasts compared to Brainvisa. A comparison between WM/GM contrast obtained for different scanners, revealed that scanner B of the 1.5 T group had significantly higher values compared to the other two scanners of the same group (A and C). This might be one reason for the disparity between the estimated volumes and GSI for centre B and those for centres A and C. However for the 3 T group, an association between GM/WM contrasts and the estimated morphometric parameters was not observed. For example, despite significantly higher GM/WM contrasts for centres F and G compared to centre H, the estimations for the three centres were mostly consistent. It should be noted however that the average WM/GM contrasts were higher in the 3 T group relative to the 1.5 T group. This therefore raises the possibility of existence of a threshold for the WM/GM contrast in order to have an effect on the morphometry results using Brainvisa.

It should be noted that the image acquisition parameters were slightly different across scanners within each group. Such disparities may add to the variability in the morphometric parameters across scanners.

As the bias correction with FAST is more robust compared to Brainvisa, it is suggested to perform the bias correction using FAST and repeat all the following steps to assess the reliabilities. This would allow the evaluation of the robustness of the following analysis steps using Brainvisa. The histogram-based approach of Brainvisa can then be compared to the Hidden Markovian Random Field model-based approach of FAST. Such a step-by-step comparison can in turn help in identifying the ways in which the program can be more robust. Currently, it is not possible to skip the bias correction step and import a bias corrected image. Instead, all steps need to be performed subsequently within the program.

Using both Brainvisa and FAST, the between-visit and between-centre reliabilities for GM and WM volumes were mostly smaller compared to the calibration study of Schnack et al. which confirms the effectiveness of using calibration factor for brain segmentation in multicentre studies.

For sulcus-specific attributes, the evaluation was performed with each of the four sulci identification algorithms so that the different algorithms could be compared. These algorithms vary in their approach in registering the brain to a 3D probabilistic atlas of the sulci (SPAM). On average, Local and Markovian algorithms predicted higher values for mean geodesic depths compared to Global and Talairach algorithms, suggesting that Local and Markovian tend to group deeper folds with the same label. In terms of reliability, for all sulcal parameters, Talairach showed lowest reliabilities whereas Markovian and Global achieved highest reliabilities. This confirms the fact that registration of different brains to Talairach atlas entails poor sulci alignment between individuals and that the other three algorithms are more successful in sulci alignment.

Nevertheless, the average reliabilities with all algorithms were mostly moderate. This clearly limits the suitability of sulcal surface and mean geodesic depth for multicentre or longitudinal studies. Further improvement may be achieved by improving the primary steps (bias correction and possibly histogram analysis) in order to obtain more reproducible estimations.

Due to the various sources of variability between the 1.5 T and 3 T groups (e.g. subjects, age, gender, field strength, number and types of scanners, and image acquisition parameters), the contribution of each source of variability to the total variance between the two groups can not be estimated. Nonetheless, a qualitative comparison suggests that while reliabilities of segmented brain volumes are higher for the 1.5 T group compared to the 3 T group (with both Brainvisa and FAST), for sulcal parameters the reliabilities are higher for the 3 T group. This suggests that fold detection and brain segmentation are not equally affected by these factors. A prospective study with various field strengths, scanner types, and image acquisition parameters on the same group of subjects would be useful for independently investigating the effect of each factor on the reliability of the results. Further investigation of the contribution of each factor, may also be useful for correcting for scanner-related variability in addition to providing information about the required criteria (image resolution, for example) for achieving more robust morphometry results and higher reliabilities with Brainvisa.

## Conclusions

In this paper we explored the consistency of brain morphometry results using Brainvisa among different scanners and different sessions of the same scanner. Our results indicate that there is occasionally considerable disparity between the values estimated for different scanners and different sessions. However, different scans of the same scanners produced more consistent results compared to those obtained with different scanners. These findings emphasize that for any kind of morphometry analysis with Brainvisa using data from multicentre or longitudinal studies, the scanner-related variability must be taken into account and where possible the resultant inconsistency should be corrected for. Furthermore, our findings provide a first step for investigation of the possibilities for improvement of Brainvisa.

## Competing interests

The authors declare that they have no competing interests.

## Authors' contributions

MS analysed and interpreted the data, drafted the manuscript and is the principal author of the manuscript. AB participated in the design, coordination, and data acquisition for the Neuro/Psygrid project and assisted in the preparation of the manuscript. JS conceived of the Neuro/Psygrid project, participated in its design and coordination of data and revised the manuscript for intellectual content. TWJM participated in the design of the CaliBrain project. DB participated in data acquisition for both Neuro/Psygrid and CaliBrain projects. DJ participated in the design and coordination of data for both Neuro/Psygrid and CaliBrain projects and revised the manuscript for intellectual content. KL participated in data acquisition for both Neuro/Psygrid and CaliBrain projects. PD participated in the design and coordination of data for the Neuro/Psygrid project and revised the manuscript for intellectual content. TRM participated in subjects' recruitment and data acquisition for the Neuro/Psygrid project. CM, SM, and SCRW participated in data acquisition for the Neuro/Psygrid project. SML conceived of both Neuro/Psygrid and CaliBrain projects, and participated in the design and coordination of data and revised the manuscript for intellectual content. BD conceived of the Neuro/Psygrid project, participated in its design and coordination of data and revised the manuscript for intellectual content. SRW participated in the design of the Neuro/Psygrid project and the coordination of data. BC participated in the design and coordination of data, revised the manuscript for intellectual content and is the senior author of the manuscript. All authors read and approved the final manuscript.

## Pre-publication history

The pre-publication history for this paper can be accessed here:

http://www.biomedcentral.com/1471-2342/11/23/prepub
